# Learning dynamics of muscle synergies during non-biomimetic control maps

**DOI:** 10.1017/wtc.2024.24

**Published:** 2025-01-20

**Authors:** King Chun Tse, Patricia Capsi-Morales, Cristina Piazza

**Affiliations:** 1 Department of Computer Engineering, School of Computation, Information and Technology, Technical University of Munich, Munich, Germany; 2 Munich Institute of Robotics and Machine Intelligence, Technical University of Munich, Munich, Germany

**Keywords:** muscle synergies, myoelectric control, prostheses

## Abstract

Advanced myoelectric prostheses feature multiple degrees of freedom (DoFs) and sophisticated control algorithms that interpret user motor intentions as commands. While enhancing their capability to assist users in a wide range of daily activities, these control solutions still pose challenges. Among them, the need for extensive learning periods and users’ limited control proficiency. To investigate the relationship between these challenges and the limited alignment of such methods with human motor control strategies, we examine motor learning processes in two different control maps testing a synergistic myoelectric system. In particular, this work employs a DoF-wise synergies control algorithm tested in both intuitive and non-intuitive control mappings. Intuitive mapping aligns body movements with control actions to replicate natural limb control, whereas non-intuitive mapping (or non-biomimetic) lacks a direct correlation between aspects, allowing one body movement to influence multiple DoFs. The latter offers increased design flexibility through redundancy, which can be especially advantageous for individuals with motor disabilities. The study evaluates the effectiveness and learning process of both control mappings with 10 able-bodied participants. The results revealed distinct patterns observed while testing the two maps. Furthermore, muscle synergies exhibited greater stability and distinction by the end of the experiment, indicative of varied learning processes.

## 1. Introduction

Individuals with limb loss face significant daily challenges. Prostheses aim to assist them in daily activities, thereby improving their quality of life and independence. Modern prostheses commonly rely on electromyographic (EMG) signals from the residual limb to control the system. Despite their potential to provide more versatile and energy-efficient replacements to body-powered prostheses, they require extensive training due to the cognitive burden, especially the more dexterous the system is. Difficulties in mastering control are a key factor in users abandoning their prostheses before becoming proficient (Salminger et al. 2022). While virtual training tools have been introduced to engage users (e.g., Armiger and Vogelstein 2008), successfully transferring myocontrol skills from virtual environments to real prosthesis use remains a significant challenge (Kanzler et al. 2019; Terlaak et al. 2015; van Dijk L et al. 2016).

Research has focused on decoding user intentions to enable simultaneous and natural control of multiple degrees of freedom (e.g., Huang et al. 2005; Smith et al. 2015), but challenges remain in successfully applying these approaches to real-world scenarios (Farina et al. 2023). Recently, high-density surface electromyography (HD-sEMG) sensors have garnered significant attention. Previous efforts have explored the application of HD-sEMG in myoelectric control algorithms (Barsotti et al. 2019; Chen et al. 2020). These sensors capture high spatial resolution signals from muscle groups, enabling detailed observation of muscle activation. Moreover, HD-sEMG demonstrates increased robustness to issues like electrode shift compared to classic myoelectric controls (Ison and Artemiadis 2014; Muceli et al. 2013).

The effortless execution of movements in healthy subjects, such as reaching, grasping, and stabilizing the body’s center of mass contradicts the intricate complexity involved in those due to the multiple muscles, joints, and bones that are engaged. Understanding how the nervous system orchestrates these actions remains a significant challenge in the field of motor control. The human motor system’s ability to coordinate several degrees of freedom (DoFs) and motor redundancy offers flexibility in executing actions. Electrophysiological studies in animals suggest that muscle activation occurs in synergistic groups rather than individual muscles (e.g., Hart and Giszter 2010; Overduin et al. 2012), referred as muscle synergies. Nonetheless, various explanations and interpretations have been proposed for the hypothesis of muscle synergies existence within motor control studies (Tresch and Jarc 2009). Recent research has extensively investigated this hypothesis, often employing statistical analyses of EMGs during behavior. One interpretation suggests that the brain modulates weighting and timing of a group of synergies to reduce the control dimensions and cognitive load (Bizzi and Cheung 2013). Another proposes that muscle synergies serve as a link between task-level objectives (e.g., stabilizing the center of mass) and execution level commands (e.g., activation of individual muscles) (Ting and McKay 2007). Therefore, synergies are not static elements; rather, they evolve through learning (Dominici et al. 2011). While their nature and developmental process remain subjects of ongoing investigation, synergies offer valuable insights into motor performance and learning conditions. Furthermore, the exploration of muscle synergies extends to myoelectric control algorithms. For instance, Choi and Kim (2011) detected four muscle synergies from wrist movements for controlling a 2D cursor, while Jiang et al. (2008) proposed an algorithm for DoF-wise synergies to estimate wrist forces. While classification techniques may yield more accurate initial prosthesis performance, the use of synergistic control methods offers potential advantages in long-term usage and adaptation to new muscle conditions.

Furthermore, establishing the relationship between user movement intentions and the reference commands for actuators, also known as maps, is a fundamental aspect of myoelectric control. While intuitive mappings aim to replicate human movement directions, non-intuitive mappings may not directly mirror user movements. The latter is also known in literature as “non-biomimetic” or “arbitrary” control maps. Ison et al. (2014) and Schone et al. (2024) emphasize the growing interest of such methods and their learning capabilities. Both mapping approaches tend to perform similarly after training (e.g., Ison and Artemiadis (2015); Radhakrishnan et al. (2008)). Additionally, subjects retain learning when using the same mapping in a new task (Antuvan et al. 2014). Despite the prevalence of intuitive mappings, identifying suitable EMG signals for these can be challenging, especially for patients with motor disabilities (Thorp et al. 2015; Verros et al. 2019). Hence, non-intuitive mapping could offer design flexibility, or other features such as under-actuation, which are especially important in the context of abnormal muscle conditions and customized solutions are needed.

In this study, we aim to assess the performance and learning dynamics of myoelectric synergistic control using two types of control mappings and employing HD-sEMG sensors. One of the main goals of this study is to compare two control approaches – intuitive and non-intuitive – both of which were tested by all participants and their relationship to a faster learning. The motor control interpretation that positions muscle synergies within a hierarchical control strategy (Lockhart and Ting 2007) suggests that they enable the organization of complex motor control variables and sensory feedback in a task relevant manner. Both aspects are pertinent to this study, as visual feedback serves as the only estimation regarding the tested mappings, given the tight relationship between estimation and control when performing a task. The two control mappings investigated possess advantages and drawbacks, potentially influencing the learning curve of new myoelectric users. We hypothesize that the majority of individuals will find the intuitive map easier to use. Nonetheless, the main focus of this research is to determine whether the ability to excel in the typically more complex map (i.e., non-intuitive) is linked to specific muscle conditions of the participants. Our previous study (Tse et al. 2023) assessed learning through performance metrics and user experience, demonstrating equivalent performance and perception across both control mappings. Notably, only participants who overall performed better with the non-intuitive mapping showed performance improvements over time. In this study, we extend this research by analyzing learning dynamics through motor development. To achieve this, we classified participants based on their overall performance preference and investigated whether prior knowledge of muscle properties can predict if a subject will eventually become proficient in a particular control map. If proven, this could enhance the selection process of myoelectric controllers for individuals with limb deficiencies who require rapid learning and effective prosthesis operation. To do so, we examine changes in muscle synergies during task completion and the use of different controllers. Additionally, we explore the similarities in synergy patterns and their correlation with performance outcomes to assess muscle predisposition for learning more complex control mappings.

## 2. Material and methods

### 2.1. Synergistic control algorithm and cursor maps

**Figure 1.**
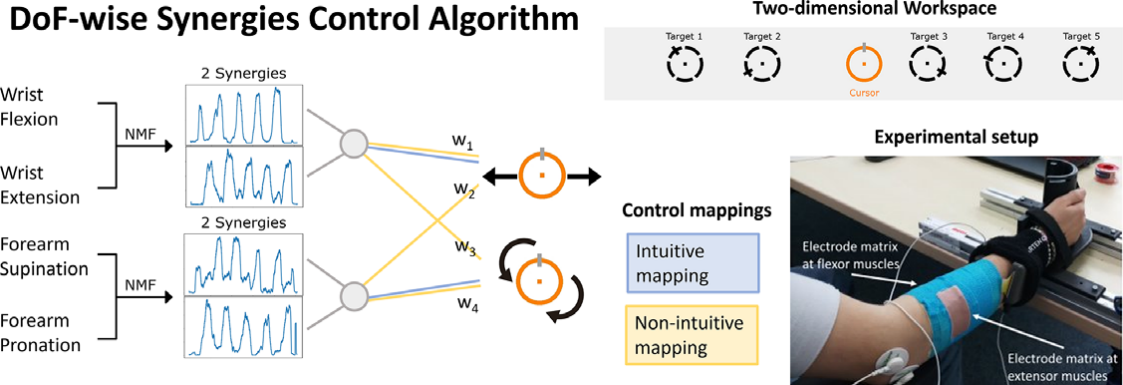
Control architecture. Electromyographic (EMG) signals are collected from the forearm area while participants performed four movements. For each pair of movements, non-negative matrix factorization is applied to the training dataset to extract four degrees-of-freedom (DoF)-wise synergies. We tested two control mappings to move a cursor in a two-dimensional workspace. The movement of the cursor for intuitive mapping is directly connected to the associated pair of synergies (blue lines). On the contrary, for the non-intuitive mapping, both pairs of synergies contribute with a certain weight 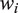 to both DoFs (yellow lines). The initial position of the orange cursor and the five targets tested are visualized in the two-dimensional workspace. On the bottom right corner, a picture of the experimental setup, with the high-density surface EMG matrices and a hand holder placed on the table.

This human study aims to assess motor learning dynamics while using a synergistic myoelectric control system. In particular, a DoF-wise synergies control algorithm was implemented for simultaneous and proportional control of the virtual prosthesis/cursor velocity (see a schematic representation in Figure 1). The proposed algorithm uses extracted muscle synergies to identify occurrence of four distinct gestures or movements. Synergies were extracted from the collected signals using non-negative matrix factorization (NMF), chosen for its robust performance across datasets (Tresch et al. 2006) and its popularity in literature for muscle synergy extraction. Two 8 × 8 high-density surface electrode grids were placed on the wrist flexor and extensor muscles. A primary synergy is extracted individually for each DoF direction of movement, as proposed in the study by Muceli et al. (2013). Wrist flexion and extension movements were categorized under one DoF, from which two corresponding synergies were extracted during a training algorithm phase of the experiment. Similarly, forearm supination and pronation movements constituted another DoF, from which two additional synergies were extracted. Each primary synergy is assumed to correspond largely to a specific gesture.

The four primary synergy weights were linked to control activation movements for the real-time control within the completion of the experiment. We evaluated the same control algorithm for two control mappings, also termed intuitive and non-intuitive. In the intuitive mappings, commands replicate human movement directions. In the non-intuitive mappings, commands may not directly mirror user movements, as all movements or both pairs of synergies contribute with a certain weight 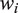 to both DoFs (see yellow lines in Figure 1). The latter introduces redundancy (i.e., under-actuated control maps), offering greater flexibility to the design. This enables navigation of the two-dimensional workspace with three commands instead of four, as first introduced in the study by Ison and Artemiadis (2015). In this work, the weights ( 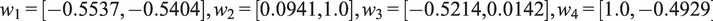 ) were selected according to Ison and Artemiadis (2015) and consistent for all subjects. Further information on the control can be found in the study by Tse et al. (2023).

### 2.2. Experimental design

Ten able-bodied participants (eight males and two females) provided informed consent and were involved in the experiment, with ages ranging from 22 to 30 years old. The study was conducted according to the guidelines of the Declaration of Helsinki, and approved by the Ethics Commission of the Technical University of Munich, reference number 478/21 S-SR (September 27, 2021). The experiment involves performing a center-out reaching task for multiple targets and testing two distinct control maps on the same day, with a break in between. Accordingly, 10 participants were divided into two equally distributed groups, testing both maps in a randomized order.

Positioned in front of a computer screen, participants rested their right forearm on a table with the hand fixed in place (Figure 1). The experiment consists of performing a two DoFs center-out reaching task using isometric muscle contractions of the wrist and forearm. The experiment started with a calibration and training algorithm phase, involving the collection of surface EMG signals to construct the control algorithm. For the pre-processing of the EMG signals, data were acquired during a no contraction or relax muscle condition, serving as the subject’s baseline. Additionally, maximum voluntary contraction (MVC) measurements were taken during the execution of the four gestures. Subsequently, data were collected for each DoF in different force levels. Participants were instructed to follow a sine wave visual cue during the intended gestures: wrist flexion/extension and forearm supination/pronation. The maximum amplitude of the sinusoidal wave corresponds to the 30% of the corresponding MVC to prevent muscle fatigue. These same values were also set as the workspace limitations of the cursor.

To train the algorithm, we first calculate the EMG envelope and the sensor bipolarity, followed by the normalization of the dataset based on the MVC and resting baseline. Each bipolar output is calculated by subtracting the signals from two consecutives EMG monopolar channels, with channel *i* subtracted from channel *i* + 1 until reaching the last row of the matrix. As a result, each HD-sEMG grid consists of 56 bipolar channels, with both grids using the same configuration. NMF was applied to the training dataset, including both extensor and flexor matrices, to extract two synergies for each DoF, corresponding to each direction of movement. During the experiment, the cursor can be directed to move left, right, rotate clockwise, or rotate counter-clockwise. Similar to conventional myoelectric controls on the market, the velocity of the cursor was proportional to the users’ synergistic muscle contractions. In the intuitive mapping, commands associated to wrist flexion and extension corresponded to moving the cursor left and right, respectively. Forearm supination and pronation movements commanded rotation in both directions. Conversely, in the non-intuitive mapping, each gesture commanded both DoFs with a specific weighting.

Participants were instructed to complete 60 trials for each mapping during the experiment, with five distinct target positions appearing randomly (see Figure 1). They were asked to hold the orange cursor on the black target for 0.5 s to successfully complete a trial, thereby assessing the controller stability. The total duration of the experiment was approximately 2 hr.

### 2.3. Metrics and data analysis

Throughout the experiment, various datasets were collected, including the raw EMG training and EMG trial data, control synergies (comprising the four synergies used for real-time control), cursor position, target position, and trial duration. The main goal of this work is to investigate distinct muscle learning dynamics corresponding to the subject overall expertise. In pursuit of this, participants were rearranged into two post hoc groups: Group IB (intuitive better) and Group NB (non-intuitive better) during data analysis. Eight participants were included in the IB group, exhibiting a higher completion rate for intuitive mapping, while two participants were included in the NB group, given their higher completion rate for non-intuitive mapping. Despite the unequal distribution of participants across the post hoc groups, this approach was necessary to evaluate distinct learning dynamics and their relation to specific muscle conditions, if any. For IB group, there were 383 successful recorded trials for intuitive mapping and 297 successful recorded trials for non-intuitive mapping. There were 120 successful recorded trials for intuitive mapping and 118 successful recorded trials for non-intuitive mapping in the NB group. Nonetheless, the number of successful trials was limited especially in temporal analysis, where each bit consisted of five trials and not all were successful. Both IB and NB groups achieved similar performance metrics when testing the intuitive mapping (Tse et al. 2023). However, significant differences were observed during non-intuitive mapping use.

#### 2.3.1. Performance analysis

This work includes a few performance metrics to study the relationship between motor learning and skill improvement, in relation to muscle conditions. In this regard, this paper elaborates on completion rate, completion time and match time. The *completion rate* is the percentage of targets successfully reached and held, divided by the total number of targets the subject could attempt using each controller. *Completion time* refers to the duration required to successfully complete a trial. *Match time* is defined as the duration from the start of the trial to the moment at which the cursor and target first align. It does not necessary indicate an immediate cursor holding on the target thereafter. Thus, the time difference between match time and completion time reflects the capacity to maintain the cursor precisely on the target position. A shorter difference indicates more stable control. The analysis of these two time metrics allows us to evaluate which grasping phases show improvement first when learning a new myoelectric controller.

#### 2.3.2. Synergies analysis

The raw EMG data collected during the trials was used for temporal analysis of motor learning. Synergies were extracted for every set of five trials, referred to as bins. To do so, the muscle activations from the initial position to the target reached were concatenated resulting in a matrix 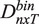 , with 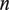 referring to the number of bipolar sEMG sensors ( 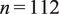 ) and 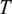 representing the total duration of the bin. A non-negative matrix factorization was applied to 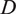 , and the optimal number of synergies was decided. We iterated from one synergy pattern up to a maximum of 10 synergy patterns to determine the optimal number of synergies corresponding to each bin. The *optimal number of synergies* is defined as the minimum number of synergies that achieves a Variance Accounted For (VAF) score greater than 85% and exhibits less than a 6% increase in the subsequent number (Israely et al. 2018). Both the quantity and morphology of synergies extracted under various experimental conditions are compared.

*Synergy similarity* ( 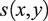 ) between two patterns is computed using cosine similarity of two vectors 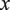 and 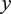 . Within a set of synergies, the similarity is determined by averaging the similarities among all possible combinations of extracted synergy patterns for a given condition or bin, excluding the diagonal. When comparing two sets of synergies, the similarity is computed based on the mean of the highest similarity combinations, known as best-matching pairs. Initially, similarities are calculated for all possible combinations, and the pair with the highest similarity is chosen to represent similar motor content. Subsequently, all other combinations involving the selected synergy pairs are disregarded. This selection process is repeated until no combinations remain, and all synergies from one dataset are matched with another synergy from the other dataset. Ultimately, the synergy similarity is determined as the average similarity between best-matching pairs of the two datasets. This procedure is employed to evaluate motor content similarity between two sets of synergies. To do so, synergies extracted from each bin with a fixed number of four were named *DoF-wise trial synergies.* They were compared to (1) the four control synergies, and to (2) those of the first bin, to evaluate synergy development across trials. Finally, the similarity procedure was employed for the comparison of best-matching pairs for (3) average synergy weights at the first and last bin corresponding to each control mapping.

## 3. Results

### 3.1. Control synergies similarity

**Figure 2.**
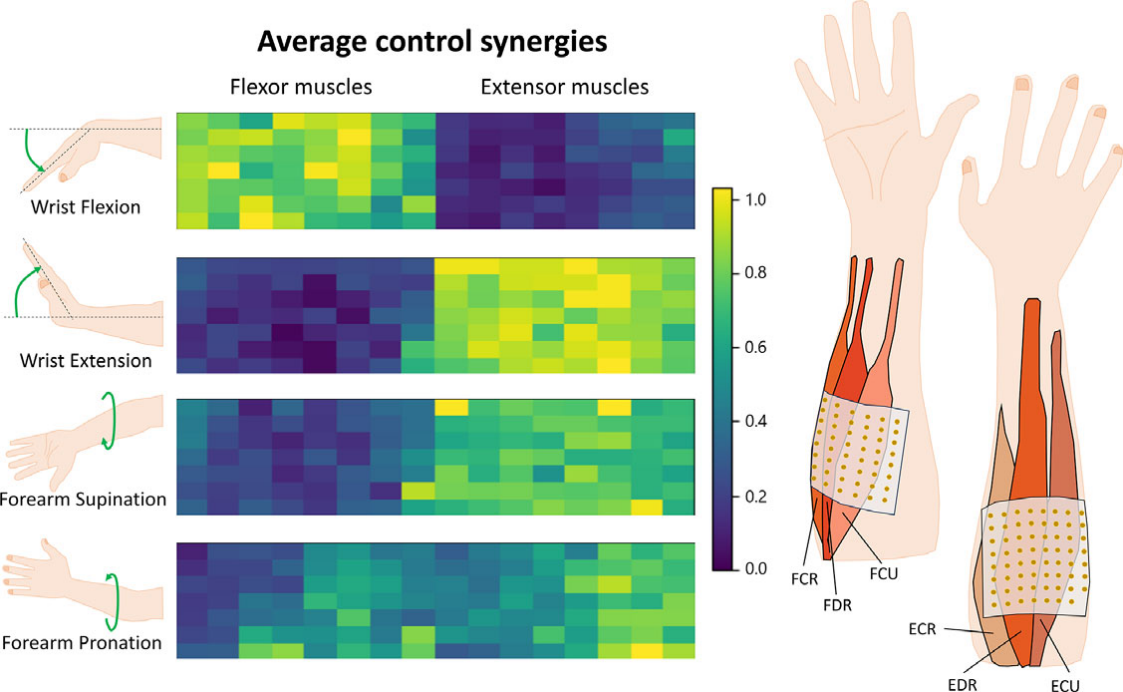
Average control synergies extracted for all subjects. Each panel displays a heatmap of the muscle synergy matrix, which is time-independent. The spatial distribution of 112 bipolar electromyography (EMG) channels is depicted with 7 rows and 16 columns per synergy. The associated movement for each synergy is indicated on the left side of the figure. On the right side, a scheme illustrating the placement and muscles involved for each high-density surface EMG matrix is provided.

The average weights extracted from all subjects, and of the four control synergies tested, are reported in Figure 2 with a heatmap representing their spatial information. During wrist flexion movement, the primary pattern is highly active over the grid placed at the wrist flexor muscles. During both wrist extension and forearm supination movements, the average primary extracted patterns show high activation over the grid placed at wrist extensor muscles. As their active spatial location overlaps, the corresponding two synergies are also highly correlated, with a cosine similarity of 0.98. The weights for the primary synergy pattern during forearm pronation movement demonstrate high activity in both wrist flexor and extensor muscles. Therefore, its active area overlaps with all three other gestures. Among them, the highest cosine similarity is 0.89 for forearm pronation and supination, followed by 0.84 with wrist extension and 0.73 with wrist flexion. Among all paired combinations, similarity is the lowest between wrist flexion and extension, with 0.38, followed by 0.52 for supination and wrist flexion.

Control synergies are defined as the four muscle patterns used as control inputs to the virtual environment. The calibration process used to extract control synergies was the same for all subjects. The subjects were then randomly assigned to two groups, according to the order mappings were tested. It is reasonable that the average control synergy similarity between these two groups followed the same distribution (Mann–Whitney *U* rank test, *p* = .690). Pearson correlation coefficient was used to quantify the linear correlation between the control synergies average similarity and the corresponding completion rate for both intuitive and nonintuitive mapping. However, no significant correlation was found for none of the control mappings. The results of all subjects for intuitive mapping showed a Pearson correlation coefficient = .326 (*p* = .358) and for non-intuitive mapping showed a Pearson correlation coefficient = −.003 (*p* = .994).

### 3.2. Performance metrics

**Figure 3.**
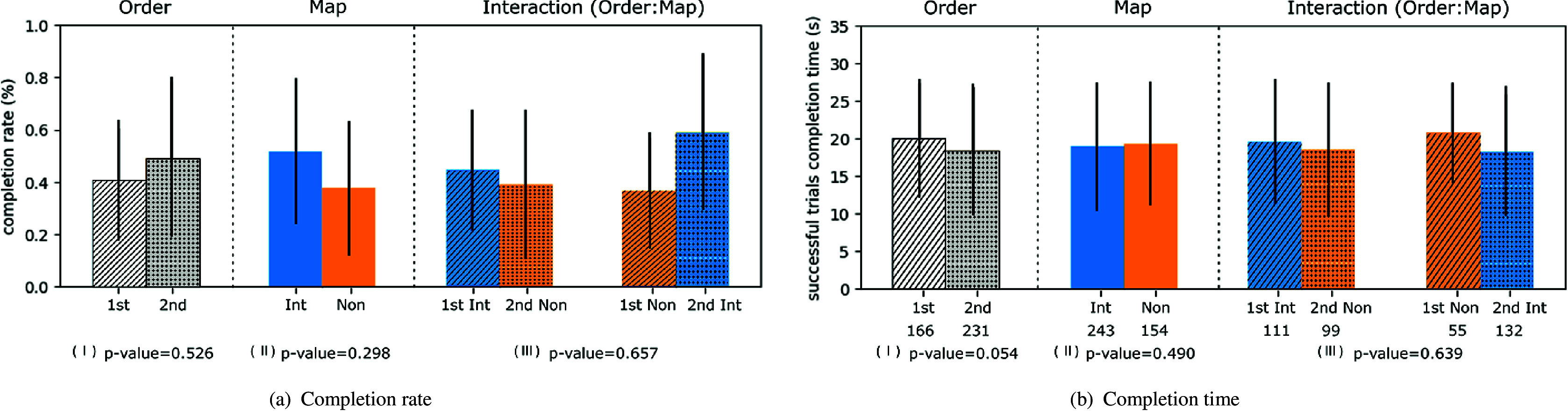
Plots of completion rate and completion time (successful trials) arranged in order, mapping and their interaction. In panel (b), the numbers on the bottom of each bar referred to the number of trials accounted for that bar. The *p*-values refer to the result of two-way ANOVA with order and mapping as factor. Note that 1st: first attempt; 2nd: second attempt; int: intuitive mapping; and non: non-intuitive mapping.

We conducted a two-way analysis of variance (ANOVA) on the completion rate and completion time (for successful trials). Neither the order nor mapping had a statistically significant effect on task performance. We present these results in Figure 3 with three plots, displaying the effects of order, mapping, and their interaction and their *p*-value in the subcaption.

**Figure 4.**
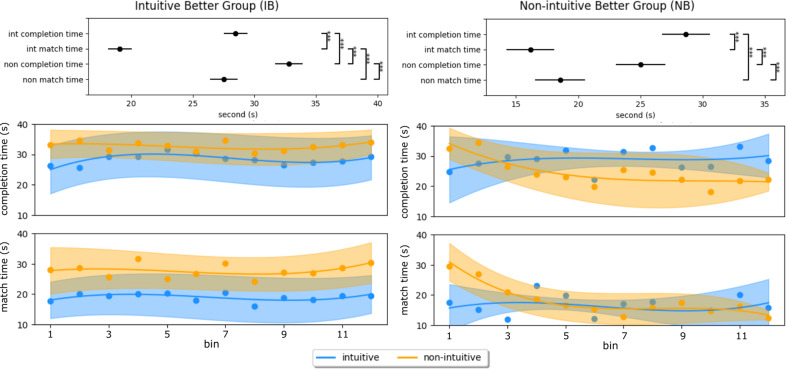
Temporal evolution of time metrics for Groups IB and NB. The top panel displays the mean estimates with comparison intervals from a Tukey’s honestly significant difference test used after significance in two-way analysis of variance (interaction). Significance among pairs is reported with asterisks. The bottom panels report the time evolution with bin averaging ± SD (bin size = 5 trials). Subjects are separated into *intuitive better* and *non-intuitive better* groups, according to their completion rate results. Note that int: intuitive mapping and non: non-intuitive mapping.

Performance results are divided post hoc to differentiate between participants that performed better for the intuitive mapping (IB), and participants who overall performed better for the non-intuitive mapping (NB). During the experiment, we noticed that participants consistently moved slightly off target. The discrepancy between match time and completion time highlighted the duration differences between the reaching and holding phases from the center-out reaching task. While these phases are relevant to our study and virtual prosthesis control, note that they pertain to the myoelectric capacity of prosthesis users. Two-way ANOVA was applied to evaluate results under these different conditions, with results reported in Table 1. Two main factors were included: *mapping* and *time metric*, with two conditions for each factor. Tukey’s honestly significant difference test was used after significance from ANOVA to observe significant differences among tested conditions. Figure 4 reported both time metrics for the two groups, testing both control mappings.

**Table 1.** Statistical results from two-way ANOVA test, for performance metrics

	Group IB		Group NB	
Source	*F*	PR (>F)	*F*	PR (>*F*)
Mapping: intuitive versus non-intuitive	123.890	<.0001	0.347	.5564
Time metric: completion time versus match time	178.432	<.0001	79.467	<.0001
Interaction	12.884	.0003	7.834	.0053

*Note.* Two groups (IB and NB) were tested separately.

Abbreviations: ANNOVA, analysis of variance; IB, intuitive better; NB, non-intuitive better.

For Group IB, both the completion time and match time of intuitive mapping were significantly smaller (*p* < .01) than that of the non-intuitive mapping. On the other hand, time metrics showed no significant difference between the two control mappings for Group NB. Comparing both metrics, during the same mapping, the match time is significantly smaller (*p* < .01) than completion time in both groups. Furthermore, the temporal evolution of completion time and match time revealed a consistent stability in the difference between them in Group IB (Figure 4). Each bin was computed at intervals of five trials, encompassing all pre-defined target combinations. As the number of trials increased, there was a decrease (i.e., a performance improvement) in completion time for the non-intuitive mapping of Group NB, also present in match time.

### 3.3. Development of synergies

**Figure 5.**
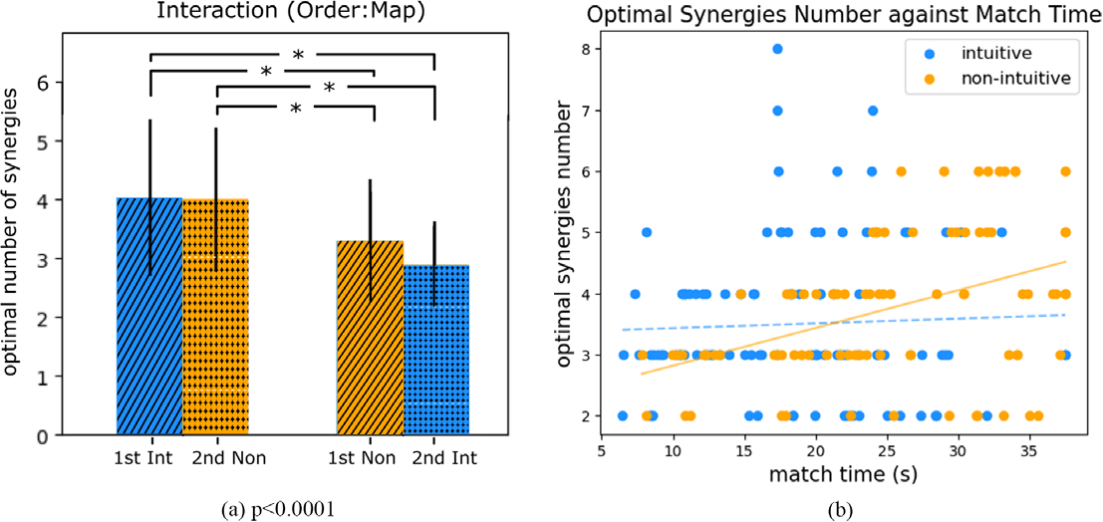
Plots of optimal number of synergies and relationship with performance. Panel (a) shows the results of two-way analysis of variance with order and mapping as factor. The *p*-value demonstrates a significant interaction between these two. Significance among pairs is reported with asterisks. Note that 1st: first attempt; 2nd: second attempt; int: intuitive mapping; and non: non-intuitive mapping. Panel (b) shows the optimal synergies numbers plotted against match time (considering all bins of the experiment). The linear regression lines of the two mappings are visualized. A solid line indicates a significant Pearson correlation. Intuitive mapping reports a Pearson correlation coefficient = .045 (*p* = .654), and non-intuitive mapping with a Pearson correlation coefficient = .418 (*p* < .0001).

The *optimal number of synergies* was then calculated offline on the data of each bin. In line with our previous analysis, we assessed the effect of order on the extracted synergies, as it cannot be assumed that the absence of a performance effect directly correlates with no impact on the developed synergies. To examine this, we conducted a two-way ANOVA and presented the interaction results for the optimal number of synergies in a bar plot in Figure 5(a). No significance was achieved in the factor order (*p* = .105, 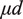 (2nd-1st) = −0.293) neither for map (*p* = .153, 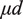 (Mon-Int) = 0.258). On the contrary, significant differences were found with the interaction of order and mapping. Participants who tried non-intuitive mapping first had smaller optimal numbers in both mappings. Furthermore, the optimal number of synergies were plotted against match time for all bins. For non-intuitive mapping, the optimal synergies number showed statistically significant linear relationship with match time with Pearson correlation coefficient = .418, *p* < .0001.

**Figure 6.**
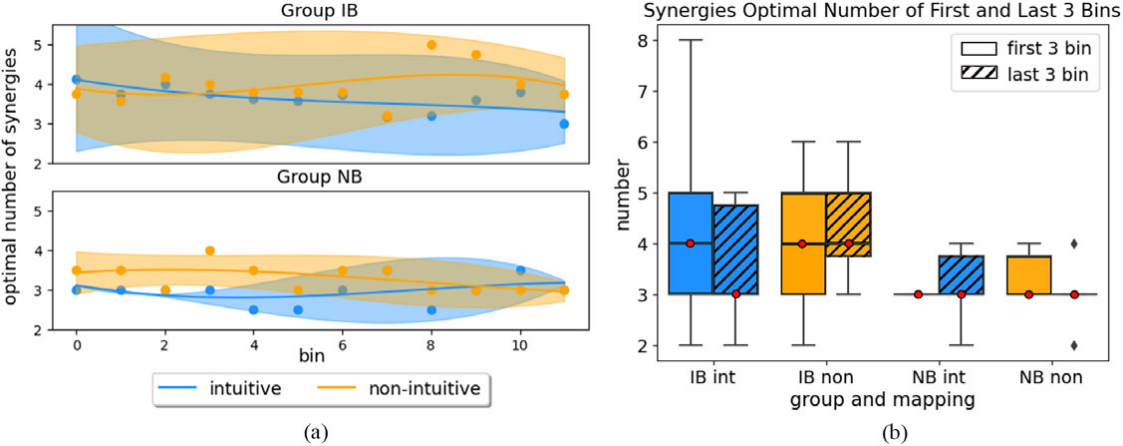
Results for the optimal number of synergies. Panel (a) reports the optimal number of synergies for each bin (bin size = 5 trials) and separated by groups. Panel (b) shows the mean (red dots) and standard deviation of optimal number of synergies for each group and mapping during the first and last 3 bins. Note that IB: intuitive better group; NB: non-intuitive better group; int: intuitive mapping; and non: non-intuitive mapping.

To assess differences in motor development according to overall performance (either IB or NB), a three-way ANOVA was used to evaluate the effect of three factors: *group*, *mapping* and *bin number*, with results reported in Table 2 (M1). *Group* is the only factor that reported significant difference, with a larger number of optimal synergies obtained for Group IB. Although without significance for interaction of factors, some trends could be observed in Figure 6(a). For Group IB, the number of synergies decreased only for intuitive mapping, and even increased by the end of the experiment in non-intuitive mapping. For Group NB, the number of synergies decreased only for non-intuitive mapping. The standard deviation among participants also decreased when comparing the optimal number of synergies between the first and last three bins, especially for Group IB (Figure 6(b)).

**Table 2.** Statistical results from three-way ANOVA test, for three different datasets highlighted in bold

	M1		M2		M3	
Source	*F*	PR (>*F*)	*F*	PR (>*F*)	*F*	PR (>*F*)
Group: IB versus NB	8.796	.004	1.730	.191	9.217	.003
Mapping: intuitive versus non-intuitive	2.204	.140	2.156	.144	2.102	.149
Bin number: 1–12	0.305	.984	0.045	1.00	3.015	.001
Group and mapping	0.004	.952	1.273	.261	1.977	.162
Group and bin number	0.333	.978	0.117	1.0	0.561	.857
Mapping and bin number	0.395	.956	0.041	1.0	0.233	.995
Group and mapping and bin number	0.255	.992	0.055	1.0	0.289	.987

*Note.* Interaction among factors is also reported. **M1:** Optimal number of synergies. **M2:** Similarity between control synergies and DoF-wise trial synergies. **M3:** Similarity between extracted synergies at first bin and DoF-wise trial synergies.

Abbreviation: DoF: degree of freedom.

**Figure 7.**
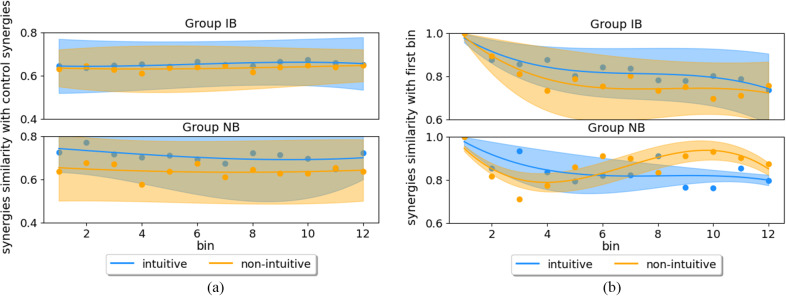
Degree of freedom-wise trial synergies similarity analysis. Panel (a) compares them to control synergies, while panel (b) to those developed at the first bin, separated in groups IB and NB. Both panels show the temporal evolution of the average synergies similarity computed for each bin (bin size = 5 trials). Note that IB: intuitive better group and NB: non-intuitive better group.

For a second analysis, four synergies were extracted for each bin and participant, named as *DoF-wise trial synergies*, and compared with the control synergies – those actually used for control during the experiments. The temporal progression of their similarity value is reported in Figure 7(a) according to the control mapping tested. The stability of the results indicates a lack of alteration in both IB and NB groups. Table 2 (M2) shows the summary of three-way ANOVA, with three factors included: *group*, *mapping*, and *bin number.*

The DoF-wise trial synergies extracted for each bin were compared to those extracted at the first bin (see Table 2, M3). Among factors, both *group* and *bin number* achieved significant difference between conditions, with PR = .003 and .001, respectively. These differences are also visible in Figure 7(b), where the temporal evolution of similarities decreased from 1.0 to around 0.8 for both control mappings in Group IB. The change in similarities was equivalent for Group NB, with a decrease to around 0.8 for intuitive mapping, already reached at bin five (after 25 reached targets). The similarity value for non-intuitive mapping decreased during the first four bins, but increased afterwards.

**Figure 8.**
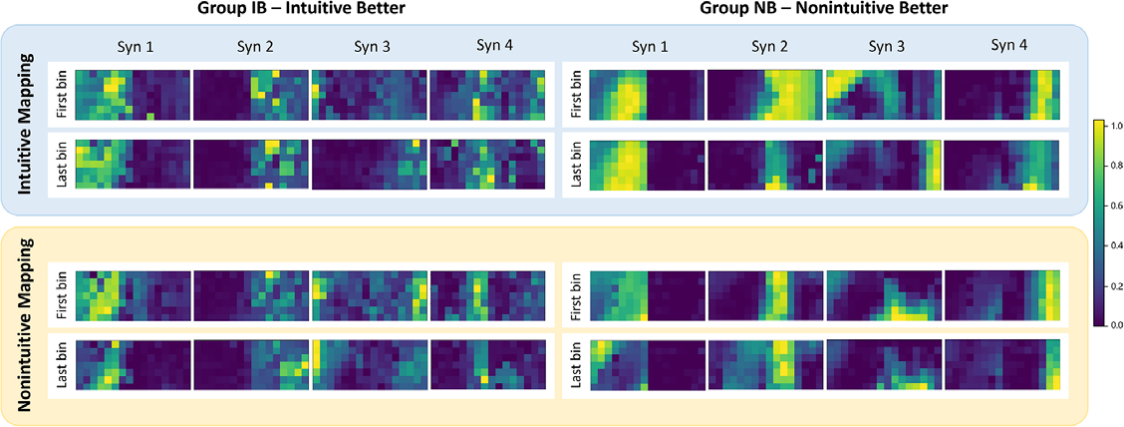
Average bin synergies weights. Synergies extracted in each group from the first and last bin and considering all subjects are reported in a heatmap to provide spatial information. Note that IB: intuitive better group and NB: non-intuitive better group.

**Figure 9.**
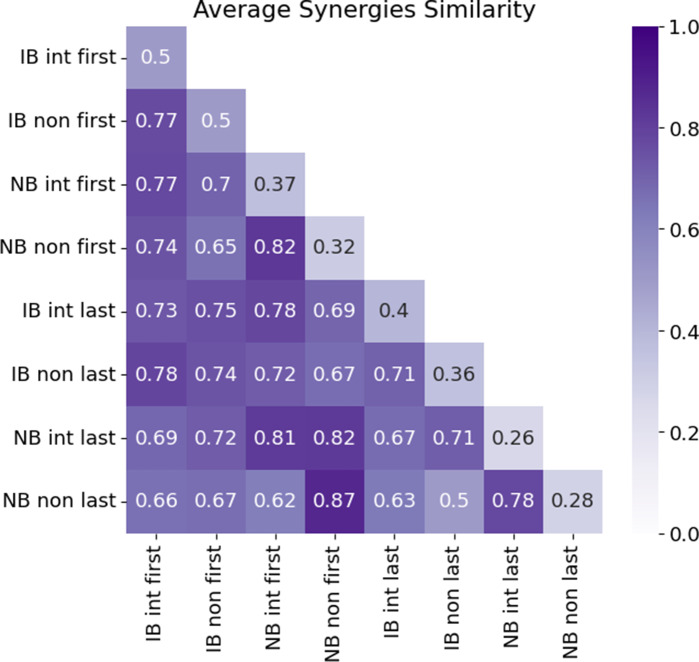
Average bin synergies similarity. The correlation heatmap displays the similarity values for first and last bin in each group. Note that IB: intuitive better group; NB: non-intuitive better group; int: intuitive mapping; and non: non-intuitive mapping; first: first bin; last: last bin.

Finally, Figure 8 reports the average DoF-wise trial synergies across all subjects, extracted at the first and last bin and divided by *group* and *mapping.* To evaluate the spatial precision of each synergy, we computed the number of highly active channels (amplitude > 0.6) in average synergies weights for the first and last bin. We achieved smaller number in all last bin cases, independently of the condition, with 15.75 channels (30.5 channels average were activated for the first bin). This indicates that there was less overlap between synergies corresponding to different movements at the end of the experiment. Note that their difference is more evident for the NB group with 36 channels first-to-last bin difference in intuitive map and 16 in non-intuitive map. For IB group, participants achieved three channels difference in intuitive map and four for non-intuitive map. Figure 9 shows that the average similarity values of last bin were slightly lower than those for the first bin, except for NB non-intuitive mapping (values at the diagonal). The similarity value was 0.78 when comparing intuitive versus non-intuitive in Group IB for both first and last bin. This indicates a consistent motor control strategy for both maps. However, this motor strategy may be only useful in one case, that is, the intuitive map. In Group NB, the value was 0.74 for first bin – intuitive versus non-intuitive, and 0.59 for last bin – intuitive versus non-intuitive.

## 4. Discussion

In this study, we assessed learning dynamics in participants using a synergistic control method and two control mappings. To train the algorithm, surface EMG signals were recorded from two muscle groups in the forearm: wrist extensors and flexors, while participants performed four distinct actions. Control synergy patterns, representing the four synergies used to command the two-dimensional workspace, were extracted for each participant before the start of the experiment. Two control mappings were tested to analyze differences in their learning curves based on the synergies developed. While under-actuation may be relevant feature for future prosthetic applications, this study primarily focuses on the foundational processes of learning non-intuitive or non-biomimetic control strategies.

The results obtained from the analysis of control synergies, as depicted in Figure 2, indicate that these synergies were not entirely distinct, as evidenced by high similarity values observed in various pairs, such as 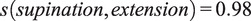 and 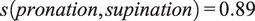 . Consequently, a wrist or forearm movement could activate multiple control synergies simultaneously, allowing the cursor to navigate in multiple directions. Participants needed to learn how to isolate single DoFs when convenient during real-time control in the center-out reaching task. In theory, a system controllability improves with more precise and distinct control synergies and a more understandable mapping. This situation enhances stability and repeatability in how a virtual prosthesis/cursor responds to each gesture command. Users can thus more rapidly identify control mapping characteristics, predict robotic system responses, and improve operational efficiency. However, no evident correlation was found between the similarity of control synergies and the completion rate, independently of the control mapping.

Regarding performance metrics, significant difference was observed between match time and completion time, indicating that subjects took considerable time to maintain the cursor on target during the holding phase, compared to the reaching phase of the trial. This trend persisted across trials, with the difference remaining consistent even as overall times decreased, as seen in Group NB for non-intuitive map. While velocity control, proportional to muscle synergies activation, enabled subjects to move the cursor toward the target more rapidly, it consistently led to overshooting. Consequently, although subjects’ intentions were accurately detected and fatigue is avoided with such method, achieving precise and stable control proved challenging. Subjects had to relax their forearm muscles to slow down cursor movement, which contrasted with the natural tendency to contract muscles when maintaining a specific arm position. Adjusting to this method and relaxing muscles quickly proved difficult for participants, particularly under stress and due to the slight delay from muscle command to visual feedback in the reference command of the cursor.

In the analysis of synergy development concerning the learning of a new motor skill, the optimal number of synergies decreased over the course of the experiment, but only for the control mapping in which subjects performed better (Figure 6(a)). The optimal number of synergies is related to the consistency of muscle activations and functional units required to execute a movement. Initially, subjects employed various muscle activation strategies to explore control options, resulting in a larger number of synergies needed. As subjects became proficient in certain gestures, they refined their motor control strategy, leading to a reduction in the optimal number of synergies and a more stable control approach by the end of the experiment. Conversely, for Group IB, the number of optimal synergies increased when testing the non-intuitive mapping (see Figure 6(a)). This suggests that even at the end of the experiment, subjects were still exploring different gestures to navigate the control mapping. Therefore, a larger number of trials would be necessary to establish an efficient control strategy for non-intuitive mapping within Group IB. In accordance, independently of the group they belong, the optimal number of synergies during the non-intuitive mapping showed a positive linear relationship with match time. Figure 5(b) shows that the smaller optimal number of synergies is related to a better control performance (i.e., shorter match time). While the reduced optimal number of synergies might seem counterintuitive regarding the use of HD-sEMG and the amount of channels recorded, these sensors provide flexibility in sensor placement and accommodate variability across subjects. Moreover, they enable recordings from deeper muscles, which can enrich the information captured. Although having perfectly positioned bipolar sensors at the belly of each muscle relevant to limb movement might not yield a significant increase in the number of necessary synergies, HD-sEMG allows for enhanced spatial visualization of the synergies (as shown in Figures 2 and 9) and provides insights into their temporal evolution concerning spatial precision.

The similarity analysis conducted on the extracted synergies (i.e., DoF-wise trial synergies) in relation to those from the first bin revealed a gradual modification in the synergies throughout the experiment. Changes within the DoF-wise trial synergies set were larger for the first four bins, which could indicate the exploration of the control mappings (Figure 7(b)). Average similarity within the best-matching pairs is more stable afterwards, which could represent the understanding and exploitation of the mapping. Despite this evolution, the changes consistently maintained a stable similarity in comparison to the four control synergies (Figure 7(a)). This indicates that new muscle synergies were formed during the experiment rather than reproducing the control synergies. However, as the myoelectric control was defined by these control synergies, these changes were constrained to ensure responsiveness of the control system.

By the end of the experiment (last bin), the developed synergies showed more localized areas of high activation within the sEMG matrix, becoming more distinguishable (Figure 9). This indicates a more precise localization of motor neuron firings corresponding to specific commands, along with enhanced motor abilities to control the virtual prosthesis/cursor. The difference in spatial active location areas between the initial and final bins is notably more pronounced in the Group NB. While this could be attributed to the complexity of the mapping and the increased motor skills required for proficiency, it is also possible that this outcome stems from the smaller sample size of participants belonging to this post hoc grouping. Despite the spatial overlap among control synergies, it is possible to develop and use new muscle synergies to independently and efficiently control each DoF, in agreement with Gigli et al. (2023).

Our results demonstrate that synergies evolved throughout the experiment in accordance with the tested control mapping. However, findings from prior research (Tse et al. 2023) suggest that synergies obtained at the end of the initial experiment (first control map tested) did not transfer motor skill to the subsequent map. Furthermore, subjects exhibited diverse synergy patterns corresponding to the tested control map. Although the number of participants in the non-intuitive better (NB) group was small, posing a significant limitation to the study, this classification was essential for identifying muscle predispositions based on individuals’ overall performance, which is the primary objective of this research. In Group NB, the similarity of synergies between the two mappings was notably lower during the final bin compared to the initial bin (Figure 9). This decrease suggests that initially, participants exhibited similar motor control strategies regardless of the implemented mapping. However, by the end of the experiment (last bin), participants in Group NB adopted different myoelectric control strategies for each corresponding mapping. Consequently, they achieved highly distinct final developed synergies for the two mappings, and demonstrated better performance with the non-intuitive mapping. Most subjects did not demonstrate significant performance improvements during the experiment, as reported in the study by Tse et al. (2023). For this reason, another limitation is the number of trials included, which may have constrained participants’ ability to fully learn the control systems. This study was motivated by the needs of prosthesis users and the investigation for simplified myoelectric control strategies suited to their limited muscle condition. However, no experiments were conducted to specifically test the generalization of these control methods to this population, which will be addressed in future research. Conducting further studies with individuals with motor disabilities would be highly valuable for assessing the applicability of such mappings in cases of limited muscle conditions. Finally, exploring a control algorithm capable of commanding a higher number of DoFs, akin to those found in modern multi-fingered prostheses, would be beneficial for evaluating motor development in these scenarios.

## 5. Conclusions

This study focuses on analyzing motor control development during the acquisition of a new skill. Specifically, it assesses changes in synergy patterns and their correlation with performance within the experiment. The developed muscle synergies offer insights into control strategies and the adaptation process. We observed an initial exploration phase within the first 20 trials, and the development of a more specialized set of synergies by the end of the experiment for both control mappings. Additionally, a few participants demonstrated the ability to interpret non-biomimetic maps and leverage their features to improve performance. In addition to muscle synergies, decomposition algorithms can be employed to extract neural information from HD-sEMG datasets (Tanzarella et al. 2021). Understanding the synergistic organization of neural inputs could provide further insights into spinal interneuron circuitry and its adaptation during motor skill acquisition. The findings of this study demonstrate that understanding muscle properties and user motor control abilities, through the use of muscle synergies, offers important insights into skill acquisition and the personalization of myoelectric control, which could be particularly beneficial for individuals with motor disabilities and limited available muscles.

## Data Availability

The authors confirm that the data supporting the findings of this study are available within the article.
